# The Clinical Examination of Sputum

**Published:** 1897-02

**Authors:** James I. Johnston

**Affiliations:** Pittsburg, Pa.


### THE CLINICAL EXAMINATION OF SPUTUM.

By JAMES I. JOHNSTON, M.D.,

Pittsburg, Pa.

It is not the intention, to present in this short article, anything very new in the clinical examination of sputum, but rather to emphasize again the use of the sputum as a factor in diagnosis to the general practitioner.

In the last few years the microscopic examination of the sputum has become of prime importance to our patients, as well as to ourselves. Since pulmonary tuberculosis, which one cannot but believe is curable in its incipient stage, kills more patients than any other disease, should we not insist as much on the examination of the sputum for suspected phthisis as we do on the thorough examination of the urine for suspected nephritis?

Let us then consider sputum examination under two heads; first, macroscopically, and second, microscopically.

The macroscopical, gross, or unaided ocular examination of sputa, should never be neglected, if possible. Patients' statements in this regard are often misleading, and, beside, to the trained physician, the objective signs are the more reliable, and generally influence more his treatment.

1. When the sputum is thin, light in color, and even streaked with blood (usually scanty), it is called mucoid, and should suggest an acute inflammation, except, perhaps, in asthma, where mucoid sputum is seen—the blood is present only in the severe cases. The frothy, watery sputum of pulmonary edema would properly come under this class.

2. As acute inflammations of the respiratory system tend to heal, to become sub-acute or chronic, the sputum becomes thicker, yellow, more viscid and abundant, and is usually spoken of as muco-purulent. The sputum of convalescing acute bronchitis, pneumonia, chronic bronchitis, and phthisis is familiar to all of us. Among the rarer forms of pulmonary affections in which this form of expectoration is found, are malignant growths in the lungs and actinomycosis—this latter can only be distinguished from chronic bronchitis by the microscope (finding the fungus). With the exception of chronic bronchitis, occasionally, the above two forms of sputum rarely have any odor.

3. Purulent sputum consists almost entirely of pus, and is slightly tenacious, yellow or green in color, fetid, and is periodically abundant. It is seen in phthisis with cavity, bronchiectasis, and would be present in abscess of lung and rupturing empyema. In hospital practice, before pulmonary tuberculosis was excluded, this periodic expectoration of purulent sputum was not an uncommon sight, for many patients did not come into the wards until late in the disease, when cavities were present.

4. Rusty and tenacious sputum always suggests croupous pneumonia, although this disease may be present without this usually characteristic sputum. This is especially true in croupous pneumonia complicating typhoid fever, and hence this condition of the lungs is frequently overlooked. It is also true in the croupous pneumonia of children and old people.

5. Again, in severe adynamic cases of croupous pneumonia, the sputum may be thin and dark colored, when it is aptly called prune-juice sputum. I have seen this a few times—once in a double croupous pneumonia complicating typhoid fever, which rapidly proved fatal. It is said to be due to blood retained in the lungs, and occurs also in gangrene and cancer of the lung.

6. Nummular sputum (so called from its coin shape) consists of round, flat, heavy masses, which sink in water. This is commonly seen in advanced phthisis.

7. Other forms are sputum containing fibrous masses; currant-jelly sputum (said to be indicative of cancer); and fetid sputum, occurring in bronchiectasis, phthisis, gangrene, abscess and occasionally in chronic bronchitis—this last separates into three distinct layers on standing.

From the rapid survey of the macroscopical appearance we turn to the second and more important division of our subject, viz.: the microscopical examination of sputum.

1. Before the work of Koch in tuberculosis, the finding of elastic tissue in the sputum was considered good evidence of phthisis, and indeed some clinicians still lay stress upon its diagnostic properties, but this should be no criterion with our present knowledge. It indicates destruction of lung tissue, but may be due to other causes than the tubercle.

It is examined as follows: The night sputum is treated to an equal part of a (20 gr. to the ounce) solution of caustic soda and boiled. As soon as it boils add about five times the amount of cold distilled water, and allow to settle. The sediment is then examined under low power as for tube casts in urine. Yellow elastic strings can then be readily seen.

2. In the sputum of asthma, and it is said also in that of croupous pneumonia occasionally, are seen spirals, named for Curschman, who first described them, which are probably molds of the finer bronchioles, and are formed of mucin. Also in the same disease, asthma, are seen transparent octohedra crystals called Charcot-Leyden crystals for these observers. These show well under the microscope and are found during and immediately after the paroxysms—their true significance is, I believe, unknown. The Charcot-Leyden crystals are found also in leucemic blood.

3. Crystals of fatty acids, and hematoidin are seen in gangrene, abscess and cancer, and indicate altered blood.

4. But the foregoing are all of minor importance to the examination of sputum for the tubercle bacillus. As Osler says, in his article on tuberculosis, and it is printed in italics: "The presence of these bacilli in the sputum is an infallible indication of the existence of tuberculosis." It is, then, the duty of every practitioner, as soon as phthisis is suspected, to examine, or to have examined, the sputum of his patient. If found in any abundance, the diagnosis is certain.

There are quite a number of methods for staining the bacilli in sputum, but the following seems to me the simplest and easiest for the use of the busy practitioner. It is a slight modification of the one taught in the laboratories of the University of Pennsylvania, a few years ago, and is, as remarked by a hospital interne, in an institution in which this method is followed, one you can almost be careless in the use of, and yet get results.

This method is as follows: A small flake or particle is selected from the specimen to be examined, and either teased and spread out on the cover glass, or perhaps better, pressed between two cover glasses, which are then separated and allowed to dry. This is then fixed by passing the cover glass a few times through the flame which coagulates the albumin. The solution of carbol-fuchsin (1—fuchsin, 10—alcohol, 100—of 5 per cent. solution of carbolic acid) is dropped upon the specimen on the cover glass, which is held over the flame until fumes are seen to rise. The cover glass is then washed with water which removes the excess of the solution, and then placed in a 5 per cent. sulphuric acid solution until the cover shows a faint pink. The counter stain is an alkaline solution of methyl blue (Lœffler's), which is then added and allowed to remain for about thirty seconds. The slide is now washed in water, dried between filter papers, mounted temporarily in glycerine, or permanently in Canada balsam, and examined. This requires a one-twelfth oil-immersion lens for satisfactory work, when the bacilli, if present, are seen stained red, while everything else in the field is stained blue. The tubercle bacillus is rod-shaped, slightly curved, about one-half the diameter of a red blood cell (which is 1-3000 of an inch) in length, with rounded ends and occasionally beaded in appearance, possibly due to spores. Its characteristic in staining is that it resists the acid differentiating solution which affects all other tissues, and hence by contrast stain we are able to see it clearly.

The thinner the film of sputum, and the more complete the differentiation, the better slide we will have for examination.

The greatest obstacle to the ready use of this examination is the possession of an oil-immersion lens on account of its expense, but I do not believe there is any one here who has not access to such a lens, either through the kindness of a brother practitioner, or a hospital laboratory. Slides could be prepared in one's office, and examined later at hospital or elsewhere in a few minutes. The absence of bacilli in one examination should not exclude the possibility of tuberculosis, but a second and even third examination should be made. The association of this bacillus with tuberculosis is beyond doubt. Since advanced by Koch, of Berlin, some twelve years ago, little has been added to, or taken from, its significance.

5. Another micro-organism in the examination of sputum, of some importance, is the diplococcus pneumoniæ of Fränkel. While this organism is often found in the sputum, even in health, it is said to be present in all expectoration of croupous pneumonia and is now, I believe, considered the cause of this disease, making the croupous pneumonia an infectious disease. In all the specimens from patients suffering from this disease, which I have examined, diplococci have been abundant. Of course the other signs and symptoms help us in the diagnosis of this disease, yet I think it is worth while looking for the bacillus, although the number present in a specimen is hardly indicative of the severity of the attack. The diplococci can be readily stained by adding to the dried sputum a solution of methyl blue, when, under a moderate power, they can be seen readily, in pairs, surrounded by a capsule, hence called diplococci.

From what has been said, it is apparent to us all that the examination of sputum, both macroscopically and microscopically, is of the utmost importance, especially the latter, for the tubercle bacillus; and that in clinical diagnosis it plays a part secondary to none, not even to the examination of the urine for albumin and casts, nor of the blood for anemia or plasmodium malariæ.

---

### THE TREATMENT OF WARTY GROWTHS OF THE GENITALS.

William S. Gottheil, in a paper on "Epithelioma of the Penis," read before the Society for Medical Progress, November 14, 1896, concludes as follows (*International Journal of Surgery*, January, 1897):

1. Warty growths of the genitals, more especially in the male, are always to be suspected of malignancy, no matter how innocent they seem.
2. They should either be left entirely alone, or be thoroughly removed by knife or cautery.
3. Inperfect attempts at destruction, as with nitrate of silver, carbolic acid, etc., are especially to be avoided; there being many cases recorded in which they have apparently stimulated a benign growth into malignant action.

